# Optimal Parameter Configuration of a Microfluidic Chip for High-Throughput, Label-Free Circulating Tumor Cell Separation and Enrichment Based on Inertial Focusing

**DOI:** 10.3390/diagnostics13233556

**Published:** 2023-11-28

**Authors:** Xiaoyi Sun, Yuqi Ma, Chunyang Lu, Ziwei Cai, Jintao Han, Zhigang Wang, Gen Yang

**Affiliations:** 1 State Key Laboratory of Nuclear Physics and Technology, School of Physics, Peking University, Beijing 100871, China; 1901110259@pku.edu.cn (X.S.); 1900011344@pku.edu.cn (Y.M.); 1701110253@pku.edu.cn (C.L.); 2101210058@pku.edu.cn (Z.C.); 1801110241@pku.edu.cn (J.H.); 2 Wenzhou Institute, University of Chinese Academy of Sciences, Wenzhou 325001, China; zgwang@ucas.ac.cn

**Keywords:** circulating tumor cell, inertial focusing, label-free, high-throughput, separation, enrichment

## Abstract

To simply, quickly, and efficiently separate circulating tumor cells from blood has always been an enormous challenge. Leveraging the principle of inertial focusing, we here designed a simply structured microfluidic chip that maintained excellent CTC separation efficiency with high robustness and low velocity sensitivity across a broad velocity range. The parameter configuration of the chip was systematically examined, especially the most influential parameters such as the arc radius and arc angle. With optimal parameters, the designed chip achieved an outstanding particle separation efficiency of 99.8% and, more importantly, enabled the efficient separation and enrichment of CTCs in blood samples. This design can be readily integrated with other functional modules for further sample processing, serving as a promising tool for cancer diagnosis and therapeutics.

## 1. Introduction

Cancer is still one of the main causes of death worldwide. Circulating tumor cells (CTCs) are considered to be a key factor involved in cancer metastasis [1]. CTCs were first discovered by Australian physician Ashworth in 1869 [2]. Due to the possibility of direct isolation of CTCs from peripheral blood, CTCs can be used as targets for ‘liquid biopsy’, providing a non-invasive method for probing metastatic cancer [3,4]. Many clinical researchers have pointed out that CTC levels in patients with metastatic cancer are associated with their clinical progression and can provide predictive and prognostic information on disease diagnosis and recurrence, overall survival, and response to therapy [5,6,7].

A major technical challenge for CTC-base liquid biopsy is that CTCs are very rare in blood, with concentrations typically estimated at a few CTCs per milliliter of whole blood among billions of erythrocytes and millions of leukocytes [8]. Therefore, how to efficiently and selectively capture CTCs is the first critical step in the analysis. For the CTC enrichment methods that have been reported so far, they are mainly divided into two categories: label-dependent methods and label-free methods [9,10].

Label-dependent methods mostly rely on specific biochemical antigens present on the CTC surface [11,12]. For example, CELLSEARCH (Raritan, NJ, USA) uses magnetic nanoparticles coated with anti-epithelial cell adhesion molecule (EpCAMs) antibodies to enrich CTCs. These methods, however, face a tough problem posed by the inherent heterogeneity in CTCs. Like most tumor tissues, CTCs are also observed to widely exhibit distinct morphological and phenotypic features [13]; for example, in order to invade blood vessels, some cancer cells may undergo an epithelial-to-mesenchymal transition (EMT), resulting in a progressive loss of expression of epithelial markers [14]. This poses a great challenge for antigen-specific CTC enrichment as no unique common antigen is available for the identification of all CTCs. In addition, these methods have other limitations, such as the permanent attachment of antigens to CTCs, which may affect the viability of CTCs and hinder subsequent analysis [15].

In comparison, label-free methods can unbiasedly separate intact, viable CTCs. These methods typically enrich CTCs by making use of their distinct physical properties, such as size, shape, deformability, adhesion, compressibility, polarizability, and magnetic susceptibility [16,17,18,19,20,21,22,23,24]. Although versatile label-free methods have been developed to date, their applications are still fettered by some limitations. A key challenge is the severe contamination of white blood cells (WBCs) in the captured CTCs due to their similar physical properties (e.g., size and shape) [25]. Methods including deterministic lateral displacement (DLD) [23,26], pinched flow fractionation (PFF) [27], and cross-flow filtration (CFF) [28] always require a large amount of buffer for processing, greatly impacting subsequent analysis; some others, such as viscoelastic microfluidics (VEM) [16], demand extra force fields or mixing with other liquids and cannot reach a high throughput. At present, there are also many methods that combine different separation methods to achieve a combination of advantages [29,30].

Of note, in contrast to other label-free methods, inertial focusing-based microfluidics (IMF) may have distinct advantages in addressing the aforementioned challenges. IMF technology operates in the medium Reynolds number range between the Stokes flow region and the turbulent flow region (1 < Re < 100). In this intermediate range, both inertial viscosity and fluid viscosity are finite and produce some interesting effects, including inertial migration and secondary flow. Accordingly, the IMF technology can achieve relatively high processing capacity, avoid cell rupture caused by excessive shear force, and separate cells only relying on inertial effects without using any additional buffer. With extremely high robustness, this technology allows much higher throughput sample processing than other methods, despite the fact that the purity of CTCs may be reduced when isolating CTCs from undiluted blood due to cell-to-cell collisions. Moreover, this technique is easy to combine with other CTC recognition methods.

In this study, utilizing the inertial focusing principle, we have designed a low-cost, high-throughput, and high-velocity sorter for CTC separation and enrichment. Because the forces that play a major role in inertial focusing are dependent on flow velocity, chip designs based on this principle are usually velocity sensitive and the available flow velocity range is relatively narrow. In this design, a series of arcs with the same angle are connected to obtain a favorable result that the focus position changes gently with the flow velocity, and a long usable flow velocity range (2–3 mL/min) is obtained and the parameters affecting the results are analyzed. Firstly, the focusing law of different sizes of particles was simulated using fluorescent microspheres as model particles. After parameter optimization, the sorter achieved highly efficient CTC separation over a large flow rate range, with significantly improved concentration and purity of CTCs and limited impact on cellular survival activity.

## 2. Materials and Methods

### 2.1. Working Principle

**Figure 1:**
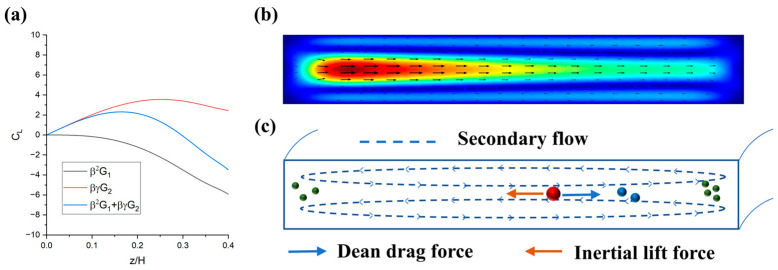
Distribution of inertial lift and secondary flow in the channel and its effect on the equilibrium position. (**a**) According to Ho’s theory [32], the inertial lift coefficient and the distribution of its two components along the axis. (**b**) The distribution of the secondary flow of the arc in a rectangular channel and (**c**) the inertial lift and Dean drag force cause particles of different sizes to be focused at different positions in the channel. Green, blue, and red represent 7.5 μm, 10 μm, and 15 μm fluorescent microspheres, respectively.

After the first observation of inertial migration [31], some experimental studies and theoretical analyses were carried out to explore the basic mechanisms of this phenomenon. Inertial lift force is considered to be a force proportional to the fourth power of the particle and changes with the position of the particle in the channel.

(1) $$F_{L}=C_{L}\frac{\rho_{f}U^{2}a^{4}}{H^{2}},$$

where $C_{L}$ is the lift coefficient, $\rho_{f}$ is the fluid density, U is the flow rate, a is the particle diameter, and H is the channel height. The distribution of $C_{L}$ can be simply deduced from the theory of Ho et al. (Figure 1a) [32] or by using the Direct Numerical Simulation (DNS) method to simulate [33,34]. Some researchers believe that there are two components of inertial lift and that the two, forces-induced lift and shear-induced lift, work together to produce an equilibrium position [35].

(2) $$F_{Ls}=\frac{\rho_{f}U^{2}a^{3}}{H},$$

(3) $$F_{Lw}=\frac{\rho_{f}U^{2}a^{6}}{H^{4}},$$

The particles within the microchannel are subjected to inertial lift force caused by a combination of shear and wall forces. The shear-induced inertial lift force caused by the parabolic velocity profile of the Poiseuille flow pushes the cells toward the wall, while the wall-induced inertial lift, which is created by the interaction of the cell and the wall, is normal to the wall surface and pushes the cells away from the wall.

Furthermore, particles within curved channels are affected by both inertial lift force *F_L_* and Dean drag force *F_D_*. This is because of the existence of secondary flow (Dean flow) in a curved channel (Figure 1b), and a dimensionless Dean number *De* is used to describe this quantity.

(4) $$De={\left(\frac{H}{2R}\right)}^{\frac{1}{2}}\cdot Re=\delta^{\frac{1}{2}}\cdot Re,$$

in which *H* and *R* represent the microchannel hydraulic diameter and curvature radius, respectively. *Re* is Reynolds number and δ is the radius of curvature.

The size of the secondary flow is considered to be proportional to the quadratic power of *De*.

(5) $$U_{D}=\frac{{De}^{2}\mu }{\rho H},$$

where μ and ρ are dynamic viscosity and density of the fluid. According to Stokes’ law, the drag force along the transverse direction on the particles can be determined by

(6) $$F_{D}=3\pi \mu U_{D}a,$$

Under the lift and drag forces, how cells are focused in curved microchannels depends on the cell diameter, concentration, flow rate, channel height, and curvature (Figure 1c).

### 2.2. Chip Fabrication

The chips were fabricated using Polymer polydimethylsiloxane (PDMS) material and soft lithography method. We used photolithography techniques to make molds on the Cr mask. SU-8 3050 was spin-coated with a spin speed of 1850 rpm at an 80 μm height. Sylgard 184 elastomer kit (Momentive Corporation, Waterford, NY, USA) with crosslinker-to-polymer ratio of 1:7 was used to make PDMS. PDMS was heated at 70 °C for more than 2 h and then peeled off the mold. Later, PDMS was bonded to glass after O_2_ plasma treatment. To avoid cell adhesion, we pre-treated the chip with 1% BSA (bovine serum albumin).

### 2.3. Preparation of Polystyrene Particle Samples

We used fluorescent particles of different sizes with polystyrene (PS) particles (from Invitrogen, Waltham, MA, USA) to mimic cells and more easily characterize the performance of the chip. Particles with a size of 7.5 μm, 10 μm, and 15 μm were used to mimic the hydrodynamic behavior of RBCs, WBCs, and CTCs (e.g., MCF-7). The concentration of particles was determined to be 1 × 10^4^/mL to evaluate the chip performance for different-sized particle separation. Particles were suspended in deionized (DI) water with 0.01% *v*/*v* Tween-20 to ensure the particles were evenly dispersed in the solution and not gathered together.

### 2.4. Cell Culture

MCF-7 cells were cultured in high-glucose Dulbecco-modified Eagle’s medium (DMEM) with 10% fetal bovine serum (FBS) and 1% penicillin–streptomycin (P/S) in a humidified atmosphere at 37 °C containing 5% (*v*/*v*) CO_2_. Before the experiment, trypsin was used to remove MCF-7 and was later suspended in DMEM.

### 2.5. Fluorescence Staining

In order to be able to better distinguish between different cells, MCF-7 cells were stained with RFP labeled with CellTracker™ Orange. WBCs in the blood were stained with Hoechst.

### 2.6. Blood Sample Preparation

Rabbit blood was used because we had easier access to it and the components are nearly the same as in humans. In total, 1 mL rabbit blood was collected into lithium heparin tubes (BD Vacutainer). To prevent coagulation, the sample was diluted with 1 mL 10 mg/mL heparin sodium solution and stored at 4 °C and processed within 4 h.

### 2.7. Chip Characterization

During the experiments, the samples were filled in an 8 mL syringe and injected into the chip through a syringe pump (Longer pump, LSP01-1BH). The flow rate for the sample injection was set as 0–3.5 mL/min. The chip was fixed on an upright microscope. The pictures were analyzed through Zen3.8, MATLAB2022a, and the ImageJ2 software.

## 3. Results

### 3.1. The Design of the Inertial Focusing Structure

There are several classic types of structures in inertial focusing: symmetrical or asymmetrical serpentine structures to focus large particles in the center of the channel and small particles on both sides of the channel [36,37,38,39], spiral structures to focus the particles at different locations near the inner side wall [40,41,42], and shrinkage and expansion structures to separate the particles of different sizes [43], as well as combinations of different structures [44,45].

**Figure 2:**
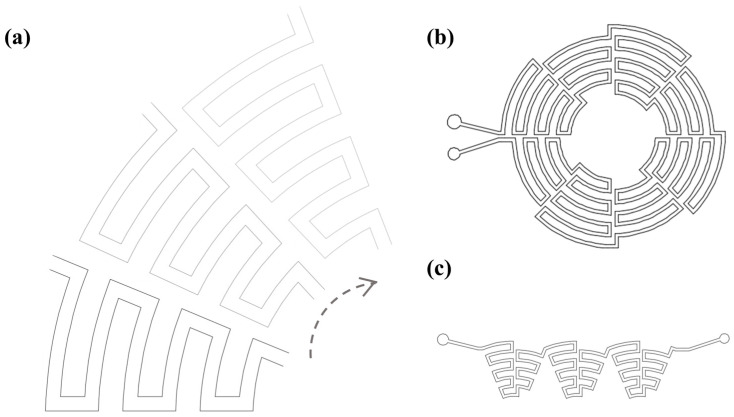
Scheme of unit cells and how they are repeated when the equilibrium distance is reached, the results are consistent across the different repetitions. (**a**) A structure that connects arcs of different radii into a unit which can be repeated to achieve the equilibrium distance. (**b**) A circular structure that shares the same center is a repetition method. (**c**) A horizontal repetition is another repetition method.

After several attempts, we found that the stable direction of the secondary flow in the arc tends to stabilize the focus of the particles, resulting in better focusing effects. However, the unstable balance of the alternating turns causes significant changes in the focus position of the particles. We connected a series of arcs with some turning in different ways, wishing to combine the advantages of a better focusing effect in the spiral channel and a more certain focusing position in the serpentine channel. It was found that a combination of good focusing and separation effects can be attained by connecting a series of arcs with the same curvature through turns and repeating this structure multiple times so as to achieve a stable equilibrium state (Figure 2a). This structure can be further either rotated to form a circle (Figure 2b) or distributed in a straight line (Figure 2c). As long as the equilibrium distance is reached (i.e., enough repeated units), the focusing and separation effect will be consistent at the outlet.

### 3.2. Particle Focusing Performance

In order to visualize the focusing status of different particle diameters, three particles with different sizes of 7.5 μm (green fluorescence), 10 μm (blue fluorescence), and 15 μm (red fluorescence) were mixed into PBS with a concentration of 10^4^/mL to obtain their position changing with flow rate (Reynolds number). After normalizing the fluorescence intensity of particles, we analyzed the maximum fluorescence brightness position (focusing position) and full width at half maxima (FWHM) at a flow rate from 0.1 mL/min to 3.5 mL/min.

**Figure 3:**
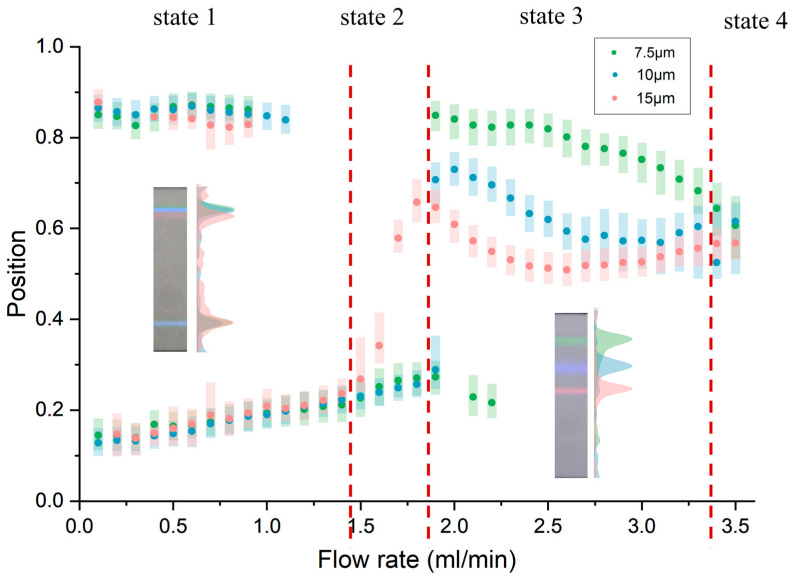
Focal of particles of different sizes as a function of flow rate.

The results showed that the focusing of particles can be divided into four different stages as the flow rate changes. At low flow rates, inertial lift plays a dominant role and the focusing effect of the chip is the same as in other designs. However, as the flow rate (Reynolds number) increases, the secondary flow gradually modifies the focusing position, causing the phenomenon to gradually differ. When the Reynolds number increases sufficiently, all particles gather in the middle and gradually defocus. The four states of focusing are shown in Figure 3.

The first state: Double focusing state. Particles of different sizes are focused on similar positions on both sides of the channel under the inertial force, which is a state suitable for liquid concentration. With the increase in flow velocity, the focusing position gradually approaches the center and changes from the double focusing state to the single focusing state.

The second state: Unstable focusing state. An intermediate state change from double-focusing to single focusing state. The particle focusing in this state is usually poor and changes greatly along with the flow rate. There are two different change modes to transform from two different focusing positions in the double focus to single focus position.

The third state: Single focusing state. Particles of different sizes continuously focus on the different positions of the channel because the Dean drag force and inertial lift are related to the particle diameter. The focusing is good and the focusing position changes slowly along with the flow rate. This state is the most suitable separation interval.

The fourth state: Defocusing state. As the Reynolds number increases, the Dean force dominates the particles, the focusing state of the particles gradually deteriorates, and particles of all sizes gather in the middle of the channel and cannot be separated.

This very long, stable separation interval of the designed system is rarely mentioned in previous reports. The weakened flow rate susceptibility greatly improves the robustness of the entire system, thus broadening its application scenarios; for example, the fluid input driver is no longer limited to sophisticated syringe pumps. Moreover, this system is also easier to combine with other structures.

### 3.3. Parametric Analysis

**Figure 4:**
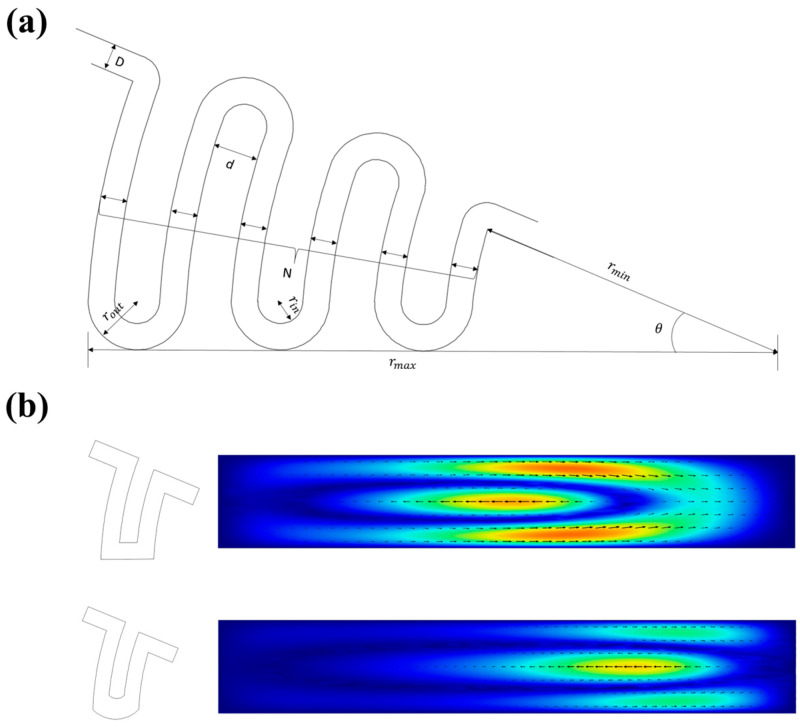
Schematic diagram of the parameters and the distribution of the secondary flow. (**a**) The multiple parameters that determine the structure can be divided into basic parameters, arc parameters, corner parameters, and channel parameters. (**b**) The influence of corner parameters on the secondary flow.

For inertial focusing, the parameters of the channel determine the focusing position. For this chip, it is obtained by multiple parameters (Figure 4a), including basic parameters (channel height ‘*h*’ and width ‘*D*’), corner parameters (inner and outer corner radii ‘*r_in_*’ and ‘*r_out_*’), arc parameters (arc radius ‘*r_min_*’, ‘*r_max_*’ and arc angle ‘θ’), channel parameters (channel interval ‘*d*’ and number ‘*N*’), etc. In order to obtain the best separation effect, we have optimized these parameters.

In order to achieve a relatively large flow rate and a stable focusing effect, the basic parameters are determined by empirical formulas, such as

(7) a/h≤0.07,

where *a* is the particle diameter and *h* is the channel height. At the same time, the aspect ratio affects the distribution of inertial lift and secondary flow. An appropriate increase in the aspect ratio can increase the usable flow velocity range. The height and width are determined to be 80 μm high and 500 μm wide. The parameters of the corner are also simulated (Figure 4b), which are not a key factor affecting the experimental results.

The arc parameters and channel parameters are not completely independent. There is a relationship between them, as follows.

(8) $$\left(N-1\right)\cdot d+N\cdot D=r_{max}-r_{min},$$

**Figure 5:**
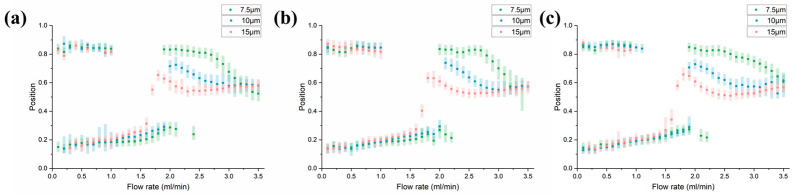
Changes in focus state as the radius increases below threshold. (**a**) *r* = 2 mm. (**b**) *r* = 3.35 mm. (**c**) *r* = 4.7 mm.

When we fix other parameters and only change the minimum arc radius, the focusing situation is shown in Figure 5. When the radius is less than a certain value, even if the minimum radius changes, the focusing position does not change much. This may be because at the same angle, a small radius has a limited action time on particles, which is closer to the connection effect of the turning.

**Figure 6:**
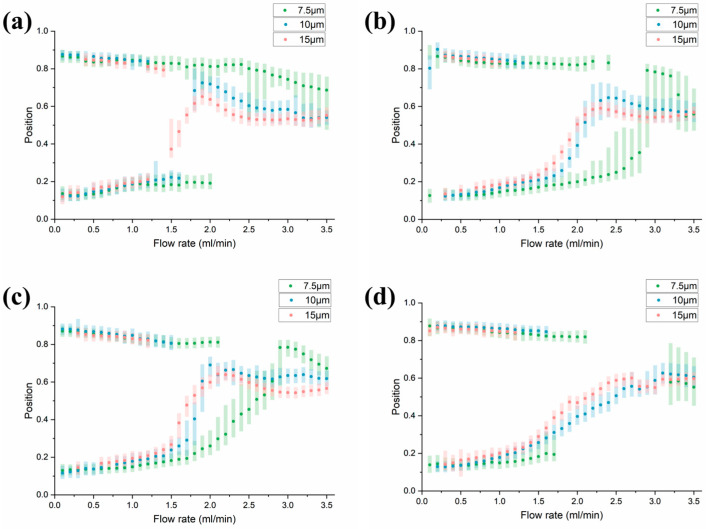
Changes in focus state as radius increases above threshold (**a**) *r* = 8.75 mm. (**b**) *r* = 10.1 mm. (**c**) *r* = 11.45 mm. (**d**) *r* = 12.8 mm.

When the radius is greater than the critical value, as it increases the focusing mode gradually changes (Figure 6). The boundary between the unstable focusing state and the single focusing state gradually blurs, and the transition from double focusing to single focusing becomes smooth. The secondary flow in the arc plays the greater role in changing the focus position, while the minimum radius determines the size of the maximum secondary flow, which has a more significant impact on the particle focusing position.

**Figure 7:**
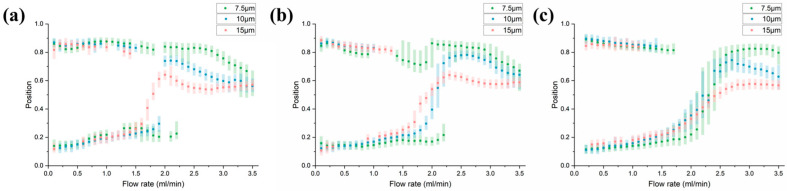
Changes in focus state as channel interval increases (**a**) *d* = 1.075 mm. (**b**) *d* = 1.86 mm. (**c**) *d* = 2.65 mm.

As the channel interval increases, the number of arcs decreases, and the modification effect of arcs on the focusing position also gradually decreases. Similar to the arc radius, the distinction between the unstable focusing state and the single focusing state gradually weakens. However, the two parameters have different effects on particles of different sizes (Figure 7).

**Figure 8:**
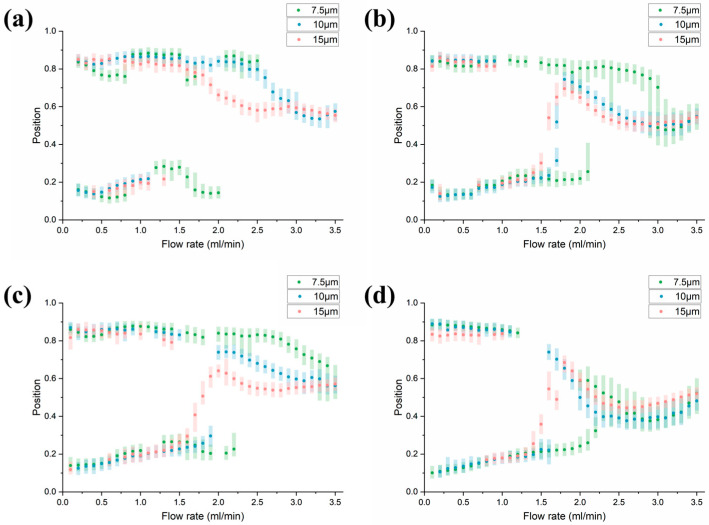
Changes in focus state as arc angle increases. (**a**) θ = 11.25°. (**b**) θ = 22.5°. (**c**) θ = 30°. (**d**) θ = 45°.

Finally, when changing the arc angle, as the angle decreases, the action time in the same arc also decreases, which slows down the modification effect on the focusing position (Figure 8), which is similar to the changes in the previous two parameters. Interestingly, although the overall impact of the three parameters on particles is similar, the impact on particles of different sizes is not the same. This allows us to choose appropriate parameter combinations to achieve the best separation effect and the longest available flow rate.

It can be seen that the arc parameter has a greater effect. It can be inferred that in an arc connected by a turn, the particles are subjectively off-center by the secondary flow and the inertial lift. The radius of the arc affects the size of the secondary flow and the angle of the arc affects the time of action, making these two parameters have the most significant impact on the result. With further parameter adjustment, we determined relatively optimal parameters, with *h* = 80 microns, *D* = 500 microns, *r_in_* = *r_out_* = ∞, *d* = 0.85 mm, *N* = 7, *r_min_* = 4.7 mm, *r_max_* = 16 mm, *θ* = 22.5°.

### 3.4. Performance Testing of the Inertial Focusing Chip

The focus position slowly changes with the flow rate and different sizes of particles always maintain different focus positions, so we have a very wide usable flow rate range (2–3 mL/min). For particles of different sizes, such as red blood cells, white blood cells, and CTCs in the blood, inertial lift and Dean drag force make them gradually separate in repeated arcs. Small red blood cells are wrapped in secondary flow and focused on both sides of the channel, and white blood cells and CTCs are focused on different off-center positions due to their subtle size differences.

**Figure 9:**
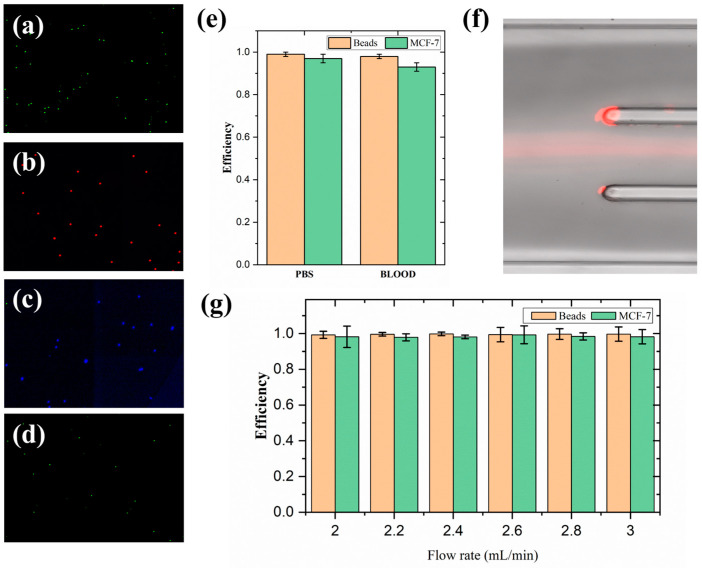
Performance of the inertial focusing chip. (**a**–**d**) Photographs of four different outlets after collection show the good separation of particles of different sizes. (**e**) Capture efficiency of 15 μm beads and MCF-7 cells at optimal flow rates in PBS and ten-fold diluted blood (*n* = 3). (**f**) The good focusing effect in the 10-fold diluted blood. (**g**) High capture efficiency was maintained at different flow rates of 2–3 mL/min (*n* = 3).

To better characterize the separation ability of the chip, we added different exports according to different needs. To separate the three different sizes of particles, we added a four-outlet design and took photos of the beads collected at the outlets, the separation efficiency for different particles reached 99.8% (*n* = 4) (Figure 9a–d).

For the application of CTC isolation, we designed a chip with three outlets according to the different sizes of CTCs and leukocytes, separating erythrocytes and leukocytes from both sides, while CTC enrichment was carried out in the middle. The 15 μm particles were used in the previous parametric analysis to simulate the size of the CTC. According to the principle of inertial focusing in the serpentine channel and the exploration of different size focusing modes in this study, particles larger than 15 μm should be focused at the focusing position near the center of the channel at a lower flow rate and stay focused in the middle. While the size of a CTC is usually more than 15 μm, 15 μm particles are used here to verify the correctness and compare the efficiency with the actual CTC. We first spiked MCF-7 cells or 15 μm fluorescent beads into PBS at a concentration of 100/mL at 2.3 mL/min; the capture efficiencies of the beads and MCF-7 were 99 ± 1% and 97 ± 2%, respectively (Figure 9e). The capture efficiency of the CTC at different concentrations was still high (5, 20, 100, 1000/mL); the average capture efficiency was 95 ± 3%. In actual patients, the number of CTCs is low, usually in a few milliliters and dozens per milliliter. This chip still maintains a high capture efficiency for a low concentration of CTCs, which can still be successfully detected for 1–10 CTCs per milliliter. This is because of the principle of inertial focusing; when the particle collision effect is excluded, that is, the lower the particle concentration, the smaller the impact on the focusing effect, the low concentration of CTC can still be focused sequentially according to the focus position. The high capture efficiency was maintained at different flow rates of 2–3 mL/min (Figure 9g).

For practical detection of blood samples, the large number of red and white blood cells in the blood will have an impact on the focusing effect, and the collision between cells has always been one of the main factors impacting the effects of inertial focusing. To simulate clinical testing, we diluted 1 mL of blood 10 times and mixed it with 15 μm fluorescent particles to demonstrate the focusing effect. As shown in Figure 9f, the beads can still maintain a focusing state, while a large number of red blood cells are separated from both sides. We also added 15 μm particles and MCF-7 cells with Hoechst staining at a density of approximately 100/mL into the chip, achieving a capture efficiency of 98 ± 1%, and 93 ± 2% in the PDMS chip (Figure 9e). White blood cells were also greatly removed during this process, which was reduced by two orders of magnitude. The number of red blood cells in the blood was too large; after separation, it had decreased by more than two orders of magnitude but there was still a large amount of residue. For the detection of CTCs, the effect of red blood cells can be removed by simple lysis. The purity of the CTCs was significantly improved, which is very beneficial for subsequent diagnoses.

## 4. Discussion

In this article, we propose a new method for separating particles of different sizes using the principle of inertial focusing. Multiple arcs of the same angle are connected through turns, and their structures are described using multiple parameters. Since the turning corners exist, particles have a focusing mode similar to a serpentine channel, and by gradually modifying the focusing position of particles through arcs, the optimal focusing and separation effect is ultimately achieved.

By dividing the parameters into different categories, the width and height of the channel are two important basic parameters which determine the flow velocity range of the inertial focusing phenomenon and the cut-off diameter that can focus the particle size. Because the inertial focusing phenomenon exists within a fixed Reynolds number range, the hydraulic diameter related to width and height will determine the actual flow velocity range, and the aspect ratio will also affect the distribution of inertial lift and secondary flow.

The parameters of the arc, including the radius and angle, which will affect the magnitude and time of the force applied in the arc, are the most important parameters that have the greatest impact on the results. The parameters of the channels, including the channel interval and number of channels, are not completely independent and indirectly affect the results. The parameters of the turning, because the particles have a shorter action time at the turning and the secondary flow distribution is relatively similar, have no significant impact on the results.

Through theoretical analysis of parameters and practical experimental analyses, we have obtained the optimal separation effect for the target particles, selected an appropriate cut-off diameter, and applied it in practical experiments. We validated the chip’s performance using MCF-7 cells and achieved very high capture efficiency not only in a PBS solution but also in blood samples. Moreover, this structure can achieve focusing and separation effects over a wide flow rate range, making it more convenient to add new structures to further improve the performance and integrate new functions.

## 5. Conclusions

Collectively, in this work, a microfluidic structure based on inertial focusing was rationally designed for particle separation. We innovatively propose a design that connects a series of arcs with the same angle through corners and achieves a good focusing effect and a definite focusing position at the same time. With optimized parameters, we found the ones that had the greatest impact on the results. The radius of the arc affects the magnitude of the drag force, and the angle of the arc affects the action time. The best separation effect of the target particles can be obtained by selecting the best parameters. This structure allows selective change in the outlet flow resistance to collect particles of different cut-off sizes. Importantly, this structure can achieve a good particle separation effect in a large flow range (2–3 mL/min) with a high particle separation efficiency up to 99.8%. When applying the structure for blood samples, the concentration and purity of CTCs were significantly increased, providing a better environment for subsequent examination and analysis.

## Data Availability

The datasets generated during the current study are available from the corresponding author upon reasonable request.
